# Shrinkage estimation of gene interaction networks in single-cell RNA sequencing data

**DOI:** 10.1186/s12859-024-05946-9

**Published:** 2024-10-26

**Authors:** Duong H. T. Vo, Thomas Thorne

**Affiliations:** Computer Science Research Centre, University of Surrey, Guildford, UK

**Keywords:** Gene network, Covariance matrix shrinkage, Single-cell RNA-seq analysis

## Abstract

**Background:**

Gene interaction networks are graphs in which nodes represent genes and edges represent functional interactions between them. These interactions can be at multiple levels, for instance, gene regulation, protein-protein interaction, or metabolic pathways. To analyse gene interaction networks at a large scale, gene co-expression network analysis is often applied on high-throughput gene expression data such as RNA sequencing data. With the advance in sequencing technology, expression of genes can be measured in individual cells. Single-cell RNA sequencing (scRNAseq) provides insights of cellular development, differentiation and characteristics at the transcriptomic level. High sparsity and high-dimensional data structures pose challenges in scRNAseq data analysis.

**Results:**

In this study, a sparse inverse covariance matrix estimation framework for scRNAseq data is developed to capture direct functional interactions between genes. Comparative analyses highlight high performance and fast computation of Stein-type shrinkage in high-dimensional data using simulated scRNAseq data. Data transformation approaches also show improvement in performance of shrinkage methods in non-Gaussian distributed data. Zero-inflated modelling of scRNAseq data based on a negative binomial distribution enhances shrinkage performance in zero-inflated data without interference on non zero-inflated count data.

**Conclusion:**

The proposed framework broadens application of graphical model in scRNAseq analysis with flexibility in sparsity of count data resulting from dropout events, high performance, and fast computational time. Implementation of the framework is in a reproducible Snakemake workflow https://github.com/calathea24/ZINBGraphicalModel and R package ZINBStein https://github.com/calathea24/ZINBStein.

## Introduction

In a biological system, specific cellular activities or functions are often carried out by interactions between genes and their products. These interactions form dynamic, complex networks which can result in changes in higher-level biological systems such as tissues and organs. Constructing and characterising these gene interaction networks are of paramount importance for guiding experimental designs, biomarker identification in diagnostic and prognostic research, target in drug discovery and development, and understanding the biological processes in an organism [1].

In gene network analysis, nodes represent genes or gene products and edges represent pair-wise relationship between them. Data from high-throughput experiments such as RNA sequencing (RNAseq) are often used to construct global gene co-expression networks which might provide insights of gene interaction networks [2]. RNA sequencing uses next-generation sequencing to quantify the amount of RNA molecules in a biological sample and reveal differences in gene expression between different samples [3]. The expression value of each gene in RNAseq data represents average gene expression of a large population of cells [4]. With advances in sequencing technologies, gene expression data are able to be extracted from individual cells in specific cell types [5]. The power and potential of single-cell RNA sequencing (scRNAseq) have been demonstrated in cell development and differentiation research [6, 7]. Computational methods to harness this potential are still in their infancy as methods developed for bulk RNA-seq data are often applied [3].

Differences in properties of scRNAseq data from bulk RNAseq data have been highlighted, including the potential for dropout events which introduce technical noise in the data, stronger overdispersion between individual cells, heterogeneity of sequenced cells due to presence of different cell populations, and different cell states and cell cycles [4]. These differences can hinder performance of existing computational tools for sequencing data analysis [3]. Therefore, computational methods specific for scRNAseq data with high performance are essential to facilitate applications of single-cell RNA sequencing in research.

In scRNAseq data analysis, the term dropout is used to describe the prevalence of excessive zero counts [8]. Dropout events are defined as the situation when expression of a gene is detected in some cells but absent in other cells of the same cell types in the same experiment [9]. Multiple factors are suggested to cause dropout events such as failure in capturing and amplifying mRNAs or limitation in sequencing depth [10]. For example, in a quantitative assessment of scRNAseq methods, a low-expressed gene TERT was only detected by quantitative polymerase chain reaction (qPCR) compared to scRNAseq [5]. Stochastic gene expression or existence of a new underlying cell subpopulation are also potential dropout-event factors [11, 12]. In the current literature, there are debates about whether dropout events occur in scRNAseq protocols using unique molecular identifiers (UMI) or whether the usage of zero-inflated modelling in scRNAseq data analysis improves performance [10, 13].

Covariance or correlation matrices from Pearson or Spearman correlation tests are used widely to generate connectivity graphs of gene co-expression. One main limitation is correlation analysis not only captures pairwise correlation between genes but also associations contributed by third-party or global effects [14]. To overcome this limit, graphical models can be applied to measure the direct interaction between genes or variables. A class of these, called Gaussian Graphical Models, make use of the partial correlation matrix, which can be calculated from the inverse covariance (precision) matrix. However, when the number of samples is comparable or smaller than the number of variables, the sample covariance matrix inherits a lot of estimation errors [15]. To solve this issue, shrinkage approaches can be adopted in which the precision matrix is assumed to be sparse.

In this study, we build on the existing Stein-type shrinkage approach of [16] by integrating a novel zero-inflated negative binomial mixture modelling approach to account for dropout in the data. We also use benchmarking approaches to identify the optimal data-transformation scheme for scRNAseq counts, and use this to construct a workflow for network inference from scRNAseq data. The main contribution of our work is the development of this workflow tailored to scRNAseq data which integrates our novel approach to accounting for dropout in the data. In our results we compare the performance and computational time of Stein-type shrinkage methods to Lasso-type shrinkage methods using simulated scRNAseq data. Finally, our suggested workflow of zero-inflated Stein-type shrinkage is applied in experimental scRNAseq data of *Schizosaccharomyces pombe*, *Saccharomyces cerevisiae*, *Plasmodium falciparum* and *Mus musculus*.

## Methods

### Overview

Graphical models are graphs in which nodes are random variables and the presence of edges represent conditional dependence or “direct links” between them [17]. Two random variables, X and Y, are considered to be independent conditionally on variable Z if their conditional probability meets:

1 $$\begin{aligned} P(X,Y|Z) = P(X|Z)P(Y|Z) \end{aligned}$$

Gaussian Graphical Models (GGMs) are a popular form of undirected graphical model in which a random variable X follows a multivariate Gaussian distribution ${\mathcal {N}}(0,\Sigma )$. The covariance matrix $\Sigma$ is unknown and nonsingular [17].

A GGM is represented by a partial correlation matrix P, as zero values in P correspond to conditional independence between variables [18]. Partial correlation coefficients measure linear relationships between pairs of random variables (*i* and *j*) which corrects for the effect from other variables (conditional dependence) [19]. The partial correlation coefficient $\rho _{ij}$ can be calculated based on $\Omega$, the inverse of covariance matrix $\Sigma$:

2 $$\begin{aligned} \rho _{ij} = -\frac{\Omega _{ij}}{\sqrt{\Omega _{ii}}\sqrt{\Omega _{jj}}}. \end{aligned}$$

In practice, the most common covariance estimator is the sample covariance matrix. However, when the number of features (p) exceeds the number of samples (n), the sample covariance matrix becomes singular and non-invertible [20]. In this situation, shrinkage techniques are often adopted to generate an invertible estimated covariance matrix for calculating the partial correlation matrix.

There are two common approaches for estimation of sparse partial correlation matrices, using $L_1$/lasso regularization in a maximum likelihood framework (Lasso-type) and linear shrinkage to shrink the sample covariance matrix towards a structured target matrix (Stein-type). In Lasso-type shrinkage, let *S* be the sample covariance matrix, and $\Theta$ be the estimated inverse covariance matrix, then the penalized log-likelihood function to maximize is [21]:

3 $$\begin{aligned} {\hat{\Theta }} = \underset{\Theta \in \mathbb {R}^{p \times p}}{argmax} \{log(det(\Theta )) - tr(S\Theta ) - \lambda \parallel \Theta \parallel _1\} \end{aligned}$$

where $\lambda$ is the amount of shrinkage applied and specified by users.

The choice of regularization parameter $\lambda$ is critical in Lasso-type shrinkage as different $\lambda$ values lead to different sparsity in the resulting graphs and therefore the scientific conclusions drawn. Multiple methods and algorithms have been developed to choose $\lambda$ which includes Akaike information criterion (AIC) and Bayesian information criterion (BIC) as standard methods for low-dimensional data. In high-dimensional data when number of features is relatively large compared to the sample size, stability approaches to regularization selection (StARS) or rotation information criterion (RIC) algorithms can be used [22, 23].

On the other hand, Stein-type shrinkage comprises three components, an empirical covariance matrix *S*, a shrinkage target matrix *T* and a shrinkage constant $\alpha$. The estimated covariance matrix is computed through a linear combination [15]:

4 $$\begin{aligned} {\hat{\Sigma }} = \alpha T + (1 - \alpha ) S \end{aligned}$$

The identity matrix is often chosen as target matrix, which leads to the shrinkage of the off-diagonal covariance coefficients towards zero, as the identity matrix is only non-zero on the diagonal of the matrix.

Similarly to Lasso-type shrinkage, the shrinkage intensity $\alpha$ controls the intensity of shrinkage. However, the value of $\alpha$ is not chosen by the user or selected using a grid search over possible values, but is derived directly by minimising a loss function which calculates the distance between the estimated covariance matrix and the population covariance matrix for a given value of $\alpha$ [24]. The equations for the optimal values of $\alpha$ are given in Table 1.

In this study, Lasso-type shrinkage is implemented with the graphical lasso (glasso) [25] and Meinshausen–Buhlmann (mb) [26] algorithms, using the huge R package [27]. Stein-type shrinkage is applied using identity matrix as target matrix and shrinkage intensity ($\alpha$) is calculated based on results of the studies [16, 28] which are named as GeneNet and Fisher2011 algorithms, respectively (Table 1, Supplementary Figure S1, S2, S3 & S4).

**Table 1:** Shrinkage intensity formula of Stein-type shrinkage

GeneNet	Fisher2011
${\hat{\alpha }} = \frac{\sum _{i\ne j}{\widehat{Var}}(s_{ij})}{\sum _{i\ne j}s_{ij}^2}$	${\hat{\alpha }} = \frac{\frac{1}{n}{\hat{a}}_2 + \frac{p}{n}{\hat{a}}_1^2}{\frac{n+1}{n}{\hat{a}}_2 + \frac{p}{n}{\hat{a}}_1^2 - 2{\hat{a}}_1 + 1}$
	$${\hat{a}}_1 = \frac{tr(S)}{p}$$
	$${\hat{a}}_2 = \frac{n^2}{(n-1)(n+2)}\frac{1}{p} \left[ tr S^2 - \frac{1}{n}(tr S)^2 \right]$$

Identity matrix is chosen as the target matrix and the optimal shrinkage intensity is derived from minimizing risk function or the Frobenius loss. The shrinkage intensity formulas from [16] (GeneNet) and [28] (Fisher2011) are used in the study

### Data transformation and p-value calculation for stein-type shrinkage workflows

In Gaussian graphical models, normality is a standard assumption about distribution of count data [29]. However, RNAseq data has specific properties such as skewness, extreme values, mean-variance dependency which deviates from the normal distribution assumption [30, 31]. Furthermore, scRNAseq can potentially deviate from a normal distribution due to other factors such as mixtures of cell subpopulations or ambient contamination. To alleviate these effects during scRNAseq data analysis, other developed methods such as cell clustering [32] to separate cells into roughly homogeneous groups to be analysed individually, and cell outlier detection can be applied in a data pre-processing step to ensure the data are amenable to graphical modelling techniques.

To improve the performance of shrinkage methods, data transformation approaches are recommended [30]. Three data transformation methods are compared in terms of enhancing performance of Stein-type shrinkage. We consider log transformation as $\log (count+1)$, which is highly used in biological data analysis to convert skew distribution towards normally distributed data [30]. We also apply a semiparametric Gaussian copula or nonparanormal transformation, which was developed for high-dimensional graphical modelling by using a Gaussian copula with Winsorized truncation [33]. This algorithm is implemented using huge.npn function from huge R package [27]. Lastly, we also consider the empirical copula $\Phi ^{-1}(\,ECDF(\,count)\,)\,$ which is similar to the nonparanormal approach and includes a Gaussian copula based on the empirical cumulative distribution of the data without truncation.

Unlike Lasso-type shrinkage methods, Stein-type shrinkage returns an estimated partial correlation matrix which requires significance testing to identify edges and generate a final graph. Without significance testing there is no way to identify non-zero entries of the partial correlation matrix from the estimated matrix, other than using heuristics based on the resulting graph structure, or by setting arbitrary thresholds, both of which could lead to large numbers of false positive predictions, especially for noisy data. Generally, partial correlation coefficients are treated as Pearson’s correlation coefficients and tested using t-statistics [34, 35]. This has been applied in the GeneNet R package for Stein-type shrinkage. To take into account the effect of shrinkage and its intensity on partial correlation matrix, Bernal et al. proposed two new probability densities for significance test which are referred to as shrunkv1 and shrunkv2 in this study [19, 35].

### Modifications in shrinkage frameworks for zero-inflated counts

To fully exploit the potential of scRNAseq data, one main challenge to overcome is the presence of excessive zeros in count data due to dropout events. Dropout events in scRNAseq data are defined as the situation where the expression of a gene is detected in some cells but absent in other cells of the same cell type [9]. Therefore, to reduce estimation errors in the sample covariance matrix and stratify biological zeros from dropout zero counts, we propose to use zero-inflated z score calculation based on zero-inflated negative binomial (ZINB) modelling of the count data in shrinkage framework. Details of the process is illustrated in Supplementary Figure S5.

In the first step, a ZINB model is fitted for expression counts of each gene *j* in cell *i* ($Y_{ij}$) by the L-BFGS-B algorithm using the optim R function to derive maximum likelihood estimates of parameters of the model:

5 $$\begin{aligned} Y_{ij} \sim w_j\delta + (1-w_j)NegativeBinomial(\mu s_i,r) \end{aligned}$$

where $\mu$ is mean, s is vector of constant scaling factors (see Supplementary Figure S5) for each cell and *r* is the size parameter of the negative binomial distribution. Parameter $w_j$ balances negative binomial model accounting for true expression counts and delta function. Importantly, the mean of the negative binomial distribution in our zero-inflated negative binomial modelling step is the product of parameters $\mu$ and scaling factors *s*. Scaling factors are commonly used in data normalization for sequencing data to account for differences in sequencing depths between samples. They are calculated using the scran R package [36].

For count of gene *j* in cell *i*, the delta function returns 0 value for all non-zero counts:

6 $$\begin{aligned} \delta (y_{ij}) = {\left\{ \begin{array}{ll} 0 & \hbox { if } y_{ij} \ne 0 \\ 1 & \text {otherwise} \end{array}\right. } \end{aligned}$$

Next, a non-detection rate ($d_{ij}$) is calculated based on the optimized ZINB model of each gene. For each count of gene j in cell i:

7 $$\begin{aligned} d_{ij} = \frac{w_j\delta (y_{ij})}{w_j\delta (y_{ij}) + (1 - w_j)NB(y_{ij};\mu ,r)} \end{aligned}$$

To select zero counts in the data resulting from dropout events, a threshold *t* with default value 0.5 is applied. After zero counts potentially resulting from dropout events are identified, a nonparanormal transformation is applied to the count data [33].

Zero-inflated z-scores are then calculated in which z-scores of zero counts identified as dropout events are set to 0. To calculate zero-inflated z-scores of nonparanormal transformed data X of gene j in cell i we use:

8 $$\begin{aligned} Z_{ij} = {\left\{ \begin{array}{ll} \mathrm {\frac{X_{ij} - {\bar{X}}_j}{\sigma _j}} & d_{ij} < t \\ 0 & d_{ij} \ge t \end{array}\right. } \end{aligned}$$

**Fig. 1:**
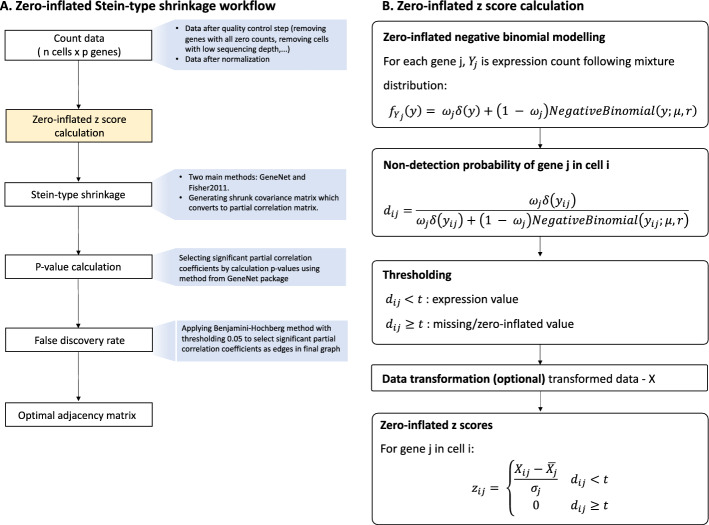
Zero-inflated covariance matrix shrinkage workflow. To account for presence of excessive zero counts in scRNAseq data, UMI count data is transformed into zero-inflated z scores for partial correlation matrix estimation. The zero-inflated z-score calculation step can be integrated in all shrinkage workflows, whereby a zero-inflated negative binomial is fitted to stratify zero counts from dropout event and “biological” zero counts. After the fitting, a non-detection rate ($d_{ij}$) is estimated in each cell for each gene. Thresholding (default t=0.5) is applied in which counts with $d_{ij}\ge t$ are considered missing or zero-inflated values. Z scores are calculated for all counts and scores of zero-inflated values are set to 0

Our proposed methodology, referred to below as ZIGeneNet, fits the ZINB mixture model given above to each gene, and then uses nonparanormal transformation of the data and the derived non-detection rates to calculate the zero-inflated z-scores $Z_{ij}$ of each count which are then used in empirical covariance matrix *S* calculation of a Stein-type shrinkage approach. The shrinkage coefficient $\alpha$ is chosen using the methodology of [16]. Significant edges are detected using correlation test statistics from fdrtool R package [34]. The workflow is outlined in figure 1.

### Performance evaluation metrics

As gene regulatory networks are sparse relative to the total possible number of edges, the task of gene regulatory network inference has a large class imbalance, with the negative class (non-edges) being much larger than the positive (edges). As a result, for the evaluation of the network inference schemes on simulated data, the Matthews correlation coefficient (MCC) is used, which is a contingency matrix method to calculate the Pearson product-moment correlation coefficient between prediction and reference values [37]. The MCC score is able to take into account the class imbalance and so gives a better measure of performance than metrics like accuracy or ROC curves. The MCC score is calculated as follows:

9 $$\begin{aligned} MCC = \frac{TP\times TN-FP\times FN}{\sqrt{(TP+FP)(TP+FN)(TN+FP)(TN+FN)}} \end{aligned}$$

where TP is the count of true positive predictions, TN is the count of true negative predictions, FP represents the count of false positives, and FN the number of false negative predictions. The MCC ranges from $-1$ to 1, which indicates the worst and perfect classification respectively. The MCC is commonly applied in binary classification evaluation for imbalanced data [37].

When benchmarking gene network inference methods it is common to rely only on synthetic data, as there is almost never a known ground truth network against which to benchmark networks inferred from experimental data. However to illustrate that the approach we propose is able to predict edges that have some supporting experimental evidence, we choose to report the numbers of predicted edges that exist in the databases listed below, both in raw figures and as positive predictive value (PPV)..

In the experimental data analysis, gene interaction databases (IntAct, STRING, BioGRID) are used as references [38–40]. In terms of gene regulatory databases, information of interactions between transcription factors(TFs) and their targets is collected from Pombase (*Schizosaccharomyces pombe*) [41], YEASTRACT (*Saccharomyces cerevisiae*) [42], and TFLink (*Mus musculus*) [43]. An integrated database of TFs and their target for *Plasmodium falciparum* is unavailable and is not included in the experimental data analysis step.

However it is important to note that the interactions in these databases are collected from multiple different experimental conditions, that are unlikely to occur simultaneously. For instance, interactions of transcription factors to initiate transcription of genes in stress response cannot be expected to be present in cells cultured in growing medium with no stress factors. Therefore, as it is not possible to reliably identify false negative edge predictions in this scenario, we only consider precision or PPV. This measures the fraction of predicted edges in the inferred network that have corresponding experimental evidence of their existence, and does not consider false negatives. PPV is calculated as follows:

10 $$\begin{aligned} PPV = \frac{TP}{TP+FP} \end{aligned}$$

## Results and discussion

### Simulating scrnaseq data from ESCO model

Simulated data for scRNAseq was generated using an algorithm based on the ESCO simulation tool [44]. A simplified simulation version with incorporated partial correlation matrix is illustrated in Supplementary Figure S5. The simplified scRNAseq model is for single cell group. Genes in the gene network are simulated to be expressed in at least 60% of cells. This matches guidelines in experimental scRNAseq data analysis in which genes not expressed in a certain number of cells are excluded for downstream analysis [45]. To simulate regulatory interactions between genes, a correlation matrix is calculated based on a partial correlation matrix provided as an input. Simulation of zero inflation in ESCO is based on mean of gene expression in which genes with higher expression mean are less likely to have zero-inflated counts [44]. This is consistent with current reports about relation between dropout event and level of gene expression [9].

### Data transformation and p-value estimation approaches in stein-type shrinkage

**Fig. 2:**
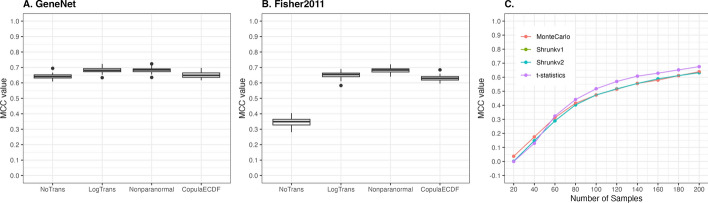
Comparative analysis of different approaches in data transformation and p-value calculations on partial correlation matrix. **A** and **B**, Three transformation methods were applied on simulated scRNAseq data with p = 200, n = 200, sequencing depth = 50,000 reads and partial correlation matrix was estimated by Fisher2011 algorithm. Compared to no transformation (NoTrans), performance of Fisher2011 was improved most by nonparanormal approach (Nonparanormal) from [33] and log transformation (LogTrans). **C** Different models to calculate p-value from shrunk partial correlation matrix were applied on simulated data (p = 200, sequencing depth = 50,000 reads, GeneNet shrinkage). Models used were t-statistics, Monte Carlo method (MonteCarlo), and shrunk probability density models for shrunk partial correlation matrices (shrunkv1 and shrunkv2 from [19] and [35], respectively)

Firstly, different methods of data transformation (pre-shrinkage step) and p-value calculation (post-shrinkage step) are explored to construct an optimal Stein-type shrinkage workflow which can be used to implement in gene network analysis of scRNAseq data. Results of the comparison between three transformation methods we consider, versus no transformation in a Stein-type shrinkage workflow when the number of genes and cells are equal to 200 and sequencing depth is 50000 reads in scRNAseq data simulation are highlighted in Fig. 2A&B. The nonparanormal approach outperforms and increases performance of Fisher2011 from MCC $\simeq$ 0.346 to MCC $\simeq$ 0.681 while MCC value is improved from 0.642 to 0.683 in GeneNet case. The effect of the three data transformation methods on scRNAseq data is visualized as histograms in Supplementary figure S6 & S7. The strong assumption of normal distribution in Fisher2011 is re-highlighted by the larger difference in performance of Fisher2011 with and without data transformation compared to GeneNet.

Secondly, performance of shrunkv1 and shrunkv2 p-value tests are compared against a significance test using t-statistics and Monte Carlo p-value calculation in scRNAseq simulated data with 200 genes. Ranging from n=60 to n=200, the t-statistics method performs best in all tested methods using GeneNet shrinkage algorithm (Fig. 2C). Therefore, nonparanormal and t-statistics approaches are used in data transformation and p-value calculation steps of the optimised Stein-type shrinkage workflow.

### Performance comparison of shrinkage methods on non-zero-inflated data

**Fig. 3:**
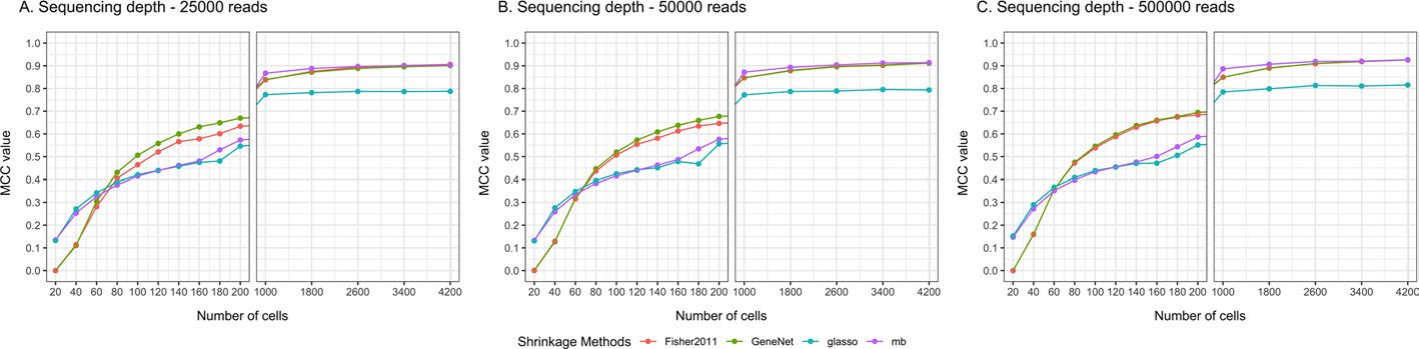
Performance of Stein-type versus Lasso-type shrinkage estimators in scRNAseq simulated data. Different sequencing depths (25,000, 50,000, 500,000 reads) were simulated in the study (iterations: 50). Used methods: GeneNet [16], Fisher2011 [28], mb - Meinshausen-Buhlmann algorithm [26], glasso - graphical lasso algorithm [25]

Performance of shrinkage algorithms are then analysed on simulated scRNAseq data, applying a nonparanormal transformation. Matthews correlation cofficient (MCC) is used for evaluation. Different sequencing depth (25,000, 50,000, 500,000 reads) are simulated and performance results are summarized in Fig. 3. We find that for low numbers of samples (n = 60 to 200) and high sequencing depth (500 000 reads), Stein-type algorithms have better performance than Lasso-type algorithms. Noticeably, Fisher2011 has slightly lower performance than GeneNet in case of 25 000 and 50 000 depths.

When designing single-cell RNA sequencing experiments, two main considerations are the number of cells and sequencing depth to sequence individual cells [46]. Increasing sequencing depth can potentially provide more information of gene transcription and reduce technical noise [47]. Interestingly, in simulation, when the number of cells is above 1000, performance of all methods approaches plateau, for example, MCC $\ge$ 0.8 at sequencing depth of 500 000 reads. To ensure the application of graphical models when the number of cells is smaller than the number of analysed genes, high-dimensional data where number of features is larger than number of observations are main focus of this study [48]. Performance of Stein-type shrinkage is also considered in another high-dimensional setting when p = 2000 and n = 20 to 200 (Supplementary Figure S8). Compared to smaller network (p=200), Stein-type shrinkage approaches retain their performance in larger network with p = 2000. At n = 100 in scRNAseq simulation, MCC of both networks (p=200 and p=2000) are around 0.5 and increases towards 0.7 when n = 200.

**Table 2:** Computational time between Stein-type and Lasso-type shrinkage algorithms (seconds)

p	n	GeneNet	Fisher2011	mb	Glasso
100	50	0.046	0.050	41.716	52.312
200	100	0.148	0.144	90.048	147.519
400	200	0.610	0.668	413.801	849.654

All analyses were performed on Macbook Pro computer with M2 chip, 8GB of RAM and MacOS operating system (10 iterations, p—number of features/genes, n—number of observations/cells)

Regarding computational cost, computing time of each algorithms is measured using workflows from Supplementary Figure S1 & S2 without a data transformation step on normally distributed data. The simulated data is constructed so that the number of cells is half of the number of genes. Results of average computational time after 10 iterations are shown in Table 2. These results highlight that Stein-type shrinkage methods can perform over 1000 times faster the Lasso-type approaches in some cases.

### Zero-inflated approach for stein-type sparse inverse covariance estimation

**Fig. 4:**
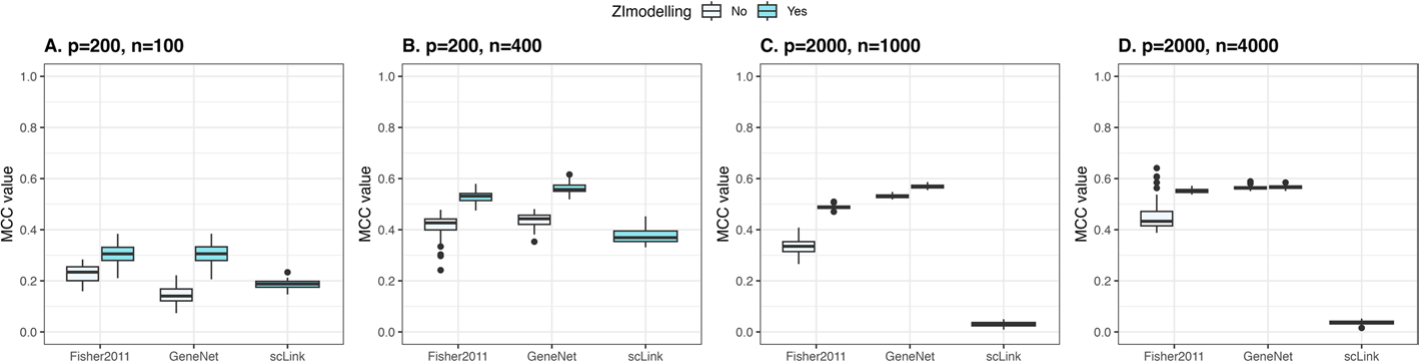
Performance of zero-inflated (ZI modelling) Stein-type and Lasso shrinkage frameworks compared to scLink workflow in zero-inflated simulated scRNAseq data (p - number of features/genes, n - number of observations/cells)

Presence of zero counts in experimental scRNAseq data of cells from the same cell type is highlighted in Supplementary Figure S9 and S10 in which gene mean is calculated based on observed/non-zero counts. The plots illustrate that genes with the same gene mean have different proportion of zero counts. This suggests that there might be a subset of genes being affected by dropouts during single-cell experiments.

**Fig. 5:**
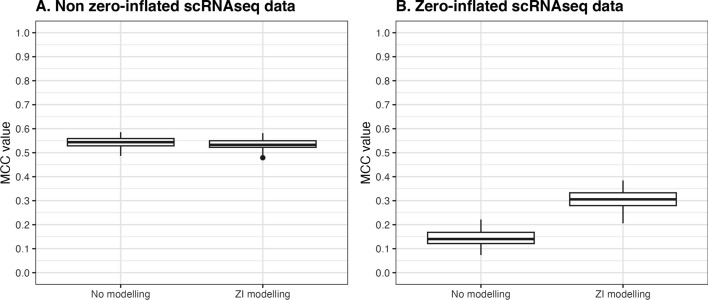
Performance with and without zero-inflated modelling in Stein-type shrinkage workflow in non-zero inflated (**A**) and zero-inflated (**B**) simulated scRNAseq data (GeneNet, p=200, n=100)

To consider the impact of modelling zero-inflation of counts for genes that are not affected by dropout, performance of the zero-inflated Stein-type shrinkage framework is tested in simulated scRNAseq data with and without zero inflation (Fig. 5 & S11). Using GeneNet and Fisher2011 with 200 genes and 100 cells, there is no significant difference in performance of ZI-integrated and non-ZI shrinkage frameworks (p-values of t-test: 0.072 and 0.443, respectively).

The zero-inflated integrated Stein-type shrinkage framework is also compared to an existing graphical model method for sparse gene co-expression network analysis of scRNAseq, scLink [49]. In summary, scLink transforms count data, and uses a Gamma-Normal mixture distribution model to detect missing-value zero counts. Pearson correlation coefficients are calculated based on high-confidence counts and used as input for Lasso-type shrinkage. The Bayesian Information Criterion (BIC) is recommended to choose the value of the regularization parameters [49]. We apply scLink using the built-in functions for data transformation and network estimation (scLink R package), and the optimal graph is chosen by selecting the smallest BIC value. Compared to our Stein-type shrinkage approach, scLink differs mainly in the zero-inflated modelling step (Gamma-Normal versus Negative binomial mixture model), and sparse inverse covariance matrix estimation step (Lasso-type versus Stein-type shrinkage estimator). Performance of scLink versus the proposed ZI shrinkage model is shown in figure 4 using ZI simulated scRNAseq data. In all the cases, GeneNet and Fisher2011 shrinkage framework taking into account zero-inflation outperforms non-ZI shrinkage workflows and scLink.

### Implementation in gene network analysis of experimental scrnaseq data

To validate interaction networks estimated from the proposed zero-inflated covariance shrinkage framework, databases curating experimental evidence of gene regulation and protein interactions from fission yeasts *Schizosaccharomyces pombe*, budding yeasts *Saccharomyces cerevisiae*, malaria parasite *Plasmodium falciparum* and house mice *Mus musculus* are used. We reiterate here that the results presented here are only intended to demonstrate how well the methods considered can recover known edges present in databases, as it is generally not possible to derive a ground truth network for a given experimental scRNAseq dataset.

For gene regulatory networks, databases of transcription factors and their targets are utilised except for *Plasmodium falciparum* [41–43]. For protein-protein interaction networks, IntAct, STRING and BioGRID are used to analysed reported interactions of gene products [38–40]. For the STRING database, only interactions having evidences in experiments and databases are considered as reported interactions.

For experimental scRNAseq data, we use high-quality count data of 108 *S.pombe* cells (strain 972 h-) [50], 127 wild-type *S.cerevisiae* cells (strain BY4741) [51], 1064 cells of ring-stage *P.falciparum* (strain DCJ) [52] and 8715 mature oligodendrocytes (MOL1 cluster) of *M.musculus* [53] are used to contruct gene regulatory networks. Genes with expression over 50 cells in each scRNAseq data set and present in interaction networks of each database are selected. Precision, or positive predictive value (PPV) is adopted as the performance metric in experimental data analysis as only a fraction of reported interactions in database are expected to be present.

**Table 3:** Comparison of experimental results with gene network databases

Data^1^	Genes	Cells	Ref^2^	Estimation methods^3^			
				ZIGeneNet	GeneNet	scLink	Pearson
[50]	570	108	TF	1/83	1/83	0/13	1/151
			Protein	728/6407	728/6407	28/935	347/4552
			All	11.4%	11.4%	3.0%	7.6%
[51]	2608	127	TF	1/116	1/109	40/11435	5599/2006048
			Protein	24/116	22/109	437/11435	118793/2006048
			All	20.7%	20.2%	4.2%	6.2%
[52]	1010	1064	TF	NA	NA	NA	NA
			Protein	48/321	52/365	30963/203718	19764/135341
			All	15.0%	14.25%	15.2%	14.6%
[53]	3860	8715	TF	0/197	0/234	56708/1422895	102516/3933970
			Protein	21/197	22/234	4018/1422895	18937/3933970
			All	10.7%	9.4%	4.2%	3.1%

Results are shown as positive predictive value, percentage or fraction between database-matching interactions and total estimated edges. Results of experimental data analysis are ordered based on size of count data. In analysis of [50, 51, 53] data, Stein-type shrinkage including ZIGeneNet and GeneNet have high precision scLink (Lasso-type shrinkage) and Pearson correlation when comparing to existing interaction databases. On the other hand, all methods perform similarly in [52] analysis

^1^Experimental scRNAseq data from *Schizosaccharomyces pombe* [50], *Saccharomyces cerevisiae* [51], *Plasmodium falciparum* [52] and *Mus musculus* [53]. Each dataset has corresponding number of selected genes and cells

^2^Reference database (All) including transcription factor (TF) database for gene regulatory interactions, protein-protein (Protein) interaction database. TF database is unavailable for *Plasmodium falciparum* in which results are shown as not applicable (NA)

^3^ZIGeneNet for GeneNet shrinkage using zero-inflated negative binomial modelling, GeneNet for GeneNet method without modification, scLink [49], Pearson for Pearson correlation

In the experimental data analysis, ZI (ZIGeneNet) and non-ZI GeneNet (GeneNet) shrinkage workflows are compared with scLink and Pearson correlation (Pearson). Pearson correlation is widely used to analyse and construct gene co-expression network [54]. The Pearson correlation matrix is calculated directly from count matrix using rcorr function from Hmisc R package [55]. Pearson correlation coefficients with Benjamini-Hochberg adjusted p-values smaller than 0.01 are selected for final graph.

The number of estimated edges which are reported in each database and total estimated edges are illustrated in table  3. Mostly, ZIGeneNet estimates fewer edges than scLink and PearCorr except on the analysis of the *S.Pombe* data [50]. In the *S.pombe* and *S.cerevisiae* scRNAseq data, the sample covariance matrices are singular as the number of genes is larger than the number of cells (570 genes versus 108 cells in *S.pombe* data and 2608 genes versus 127 cells in *S.cerevisiae* data). In these cases, Stein-type shrinkage approaches including ZIGeneNet and GeneNet have higher precision compared to scLink and Pearson methods. Specifically, around 20% of estimated edges from ZIGeneNet and GeneNet have been confirmed by other experiments in the database for *S.cerevisiae*, while positive predictive values are 4.2% and 6.2% using scLink and Pearson methods, respectively. However the scLink and Pearson methods predict more edges, so may be preferred if the goal is detecting as many edges as possible with less concern for precision. In the *P.falciparum* scRNAseq dataset, the number of cells is relatively large compared to the number of genes (1064 cells versus 1010 genes), and performances of all methods are similar, with approximately 15% precision.

To demonstrate the potential of graphical models in large-scale network inference, we apply Stein-type shrinkage workflows, scLink and Pearson correlation matrix in scRNAseq data from the mouse brain. In the study of [53], harvested brain cells are clustered and annotated using reported biomarkers. To reduce the heterogeneity in cells, network estimation methods were applied on MOL1 cluster which is a subtype of mature oligodendrocytes and has been validated from a different study [56]. Compared to scLink and Pearson correlation methods, Stein-type shrinkage workflows (ZIGeneNet and GeneNet) outperform in term of precision, although scLink and Pearson correlation predict a larger number of edges.

**Fig. 6:**
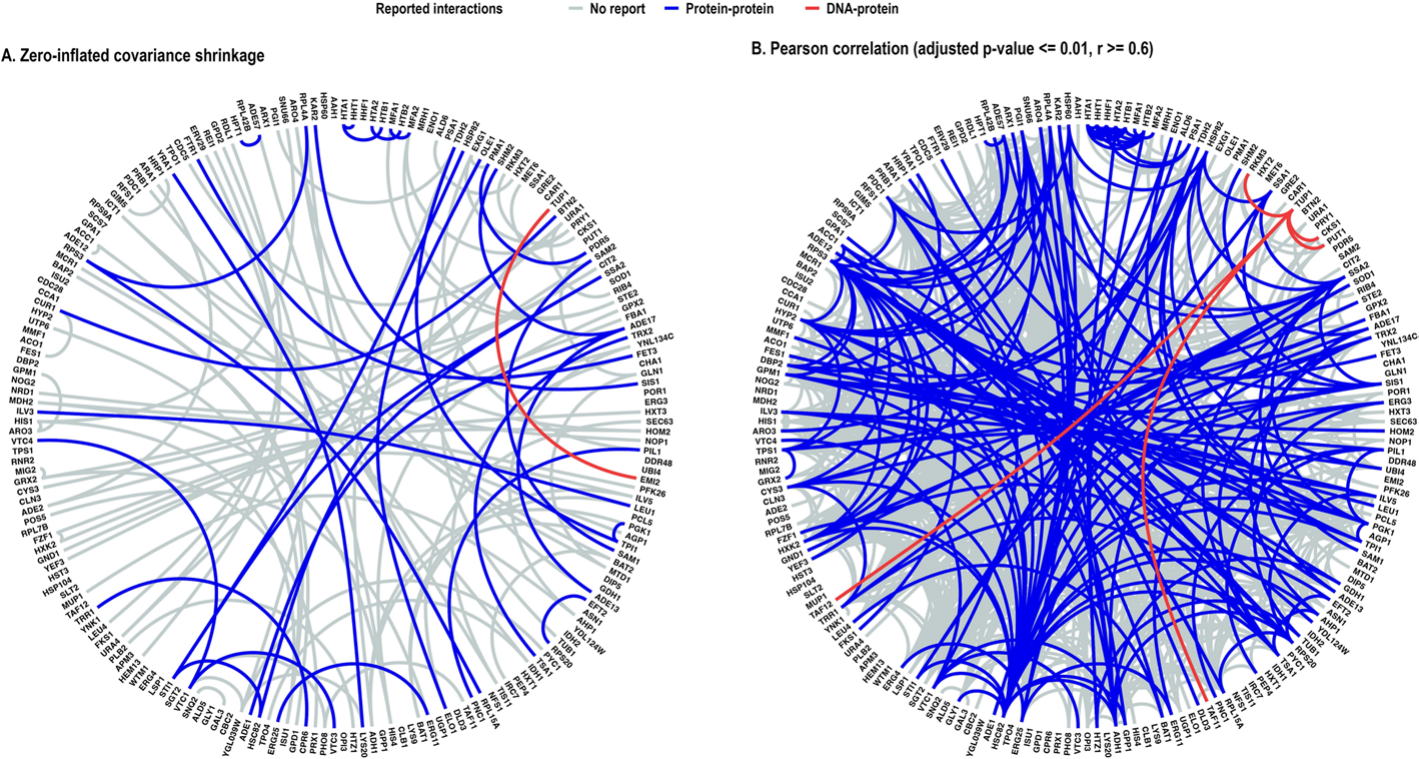
Estimated networks from zero-inflated covariance shrinkage workflow (ZIGeneNet) versus Pearson correlation workflow with coefficients (r) over 0.6 on 200 highly variable genes of *S.cerevisiae* scRNAseq data. Edges reported in databases are highlighted in red for transcription factor and gene target interactions, and in blue for protein-protein interactions

To demonstrate the impact of covariance matrix shrinkage on network sparsity, the four network inference methods (ZIGeneNet, GeneNet, scLink, and Pearson) were applied to 200 highly variable genes (HVGs) from the *S.cerevisiae* scRNAseq data of [51]. The result is shown in table S1 in which ZIGeneNet has highest precision ($\simeq$ 30%) compared to 12% precision in other methods. Estimated networks of this analysis from ZIGeneNet and Pearson were visualized in figure 6 for comparison. It can be seen that the network inferred by ZIGeneNet is considerably sparser that that of the Pearson correlation approach.

## Conclusions

Gene regulatory network inference algorithms and allow the extraction of network information from single-cell gene expression data that can be used to further our understanding of molecular networks and potentially act as a screening step to select potential proteins for downstream analysis.

We demonstrate that our proposed approach that uses a network inference method specific to scRNAseq data gives higher performance on simulated data, and when applied to experimental scRNAseq data recovers more interactions that have supporting evidence of their existence present in the databases used, than those that do not explicitly model zero-inflation. We also show that by using Stein type shrinkage and a negative binomial model of scRNAseq data that takes into account zero-inflation of counts, we can improve performance over the previously developed scLink methodology.

Due to the computational speed of Stein type shrinkage approaches, in future work we will explore the possibility of performing large numbers of local network inference tasks in scRNAseq data, to make this approach applicable to data where there are potentially continuous trajectories of cell types present, such as in developmental studies [57]. We also plan to investigate the possibility of including prior information in the target matrices of the shrinkage schemes proposed, so that known edges from databases can be integrated into the analysis. The Stein-type shrinkage method can also integrate with other sequencing analysis methods which can extend its application to heterogeneous samples when multiple cell types exists. For instance, in single-cell sequencing study of malaria parasites, cell clustering shows distinct cell clusters consistently with distinct stages of cell collection [52]. With the ability to recover different cell groups in biological samples, cell clustering can facilitate Stein-type shrinkage to implement local network inference analysis in one cluster in which most of cells are from the same cell group.

## Supplementary Information

Supplementary material 1

## Data Availability

The simulation and benchmarking code used to prepare the manuscript is available as a Snakemake workflow https://github.com/calathea24/ZINBGraphicalModel, and an R package implementing the method is also available https://github.com/calathea24/ZINBStein. Data used are publicly available from the Gene Expression Omnibus (GSE122392), NCBI Sequence Read Archive (SRP116718, SRP135960) and ArrayExpress (E-MTAB-6825).

## References

[1] Emmert-Streib F, Dehmer M, Haibe-Kains B. Gene regulatory networks and their applications: understanding biological and medical problems in terms of networks. Front Cell Dev Biol. 2014;2:38. 25364745 10.3389/fcell.2014.00038 PMC4207011

[2] Ballouz S, Verleyen W, Gillis J. Guidance for RNA-Seq co-expression network construction and analysis: safety in numbers. Bioinformatics. 2015;31(13):2123–30. 25717192 10.1093/bioinformatics/btv118

[3] Wang T, Li B, Nelson CE, Nabavi S. Comparative analysis of differential gene expression analysis tools for single-cell RNA sequencing data. BMC Bioinf. 2019;20(1):1–16. 10.1186/s12859-019-2599-6 PMC6339299 30658573

[4] Yu X, Abbas-Aghababazadeh F, Chen YA, Fridley BL. Statistical and bioinformatics analysis of data from bulk and single-cell RNA sequencing experiments. Transl Bioinf Therap Dev. 2021;143–75. 10.1007/978-1-0716-0849-4_9 PMC7771369 32926366

[5] Wu AR, Neff NF, Kalisky T, Dalerba P, Treutlein B, Rothenberg ME, Mburu FM, Mantalas GL, Sim S, Clarke MF, et al. Quantitative assessment of single-cell RNA-sequencing methods. Nat Methods. 2014;11(1):41–6. 24141493 10.1038/nmeth.2694 PMC4022966

[6] Saliba A-E, Westermann AJ, Gorski SA, Vogel J. Single-cell RNA-Seq: advances and future challenges. Nucleic Acids Res. 2014;42(14):8845–60. 25053837 10.1093/nar/gku555 PMC4132710

[7] Hedlund E, Deng Q. Single-cell RNA sequencing: technical advancements and biological applications. Mol Aspects Med. 2018;59:36–46. 28754496 10.1016/j.mam.2017.07.003

[8] Sarkar A, Stephens M. Separating measurement and expression models clarifies confusion in single-cell RNA sequencing analysis. Nat Genet. 2021;53(6):770–7. 34031584 10.1038/s41588-021-00873-4 PMC8370014

[9] Qiu P. Embracing the dropouts in single-cell RNA-Seq analysis. Nat Commun. 2020;11(1):1169. 32127540 10.1038/s41467-020-14976-9 PMC7054558

[10] Jiang R, Sun T, Song D, Li JJ. Statistics or biology: the zero-inflation controversy about SCRNA-Seq data. Genome Biol. 2022;23(1):1–24. 35063006 10.1186/s13059-022-02601-5 PMC8783472

[11] Kim JK, Kolodziejczyk AA, Ilicic T, Teichmann SA, Marioni JC. Characterizing noise structure in single-cell RNA-Seq distinguishes genuine from technical stochastic allelic expression. Nat Commun. 2015;6(1):8687. 26489834 10.1038/ncomms9687 PMC4627577

[12] Jindal A, Gupta P, Jayadeva, Sengupta D. Discovery of rare cells from voluminous single cell expression data. Nat Commun. 2018;9(1):4719. 30413715 10.1038/s41467-018-07234-6 PMC6226447

[13] Kim TH, Zhou X, Chen M. Demystifying “drop-outs’’ in single-cell UMI data. Genome Biol. 2020;21(1):196. 32762710 10.1186/s13059-020-02096-y PMC7412673

[14] Huang S, Li J, Sun L, Ye J, Fleisher A, Wu T, Chen K, Reiman E, Initiative ADN, et al. Learning brain connectivity of Alzheimer’s disease by sparse inverse covariance estimation. Neuroimage. 2010;50(3):935–49. 20079441 10.1016/j.neuroimage.2009.12.120 PMC3068623

[15] Ledoit O, Wolf M. Honey, i shrunk the sample covariance matrix. UPF economics and business working paper 2003;(691).

[16] Schäfer J, Strimmer K. A shrinkage approach to large-scale covariance matrix estimation and implications for functional genomics. In: Statistical applications in genetics and molecular biology, vol. 4(1), 2005; 10.2202/1544-6115.1175 16646851

[17] Giraud C. Introduction to high-dimensional statistics. New York: CRC Press; 2021.

[18] Whittaker J. Graphical models in applied multivariate statistics. Chichester: Wiley Publishing; 2009.

[19] Bernal V, Bischoff R, Guryev V, Grzegorczyk M, Horvatovich P. Exact hypothesis testing for shrinkage-based gaussian graphical models. Bioinformatics. 2019;35(23):5011–7. 31077287 10.1093/bioinformatics/btz357 PMC6901079

[20] Ledoit O, Wolf M. The power of (non-) linear shrinking: a review and guide to covariance matrix estimation. J Financ Economet. 2022;20(1):187–218.

[21] Banerjee O, El Ghaoui L, d’Aspremont A. Model selection through sparse maximum likelihood estimation for multivariate gaussian or binary data. J Mach Learn Res. 2008;9:485–516.

[22] Liu H, Roeder K, Wasserman L. Stability approach to regularization selection (stars) for high dimensional graphical models. In: Advances in neural information processing systems, vol. 23, 2010;. PMC4138724 25152607

[23] Lysen S. Permuted inclusion criterion: a variable selection technique. Publicly accessible Penn Dissertations, 28, 2009;

[24] Ledoit O, Wolf M. Improved estimation of the covariance matrix of stock returns with an application to portfolio selection. J Empir Financ. 2003;10(5):603–21.

[25] Friedman J, Hastie T, Tibshirani R. Sparse inverse covariance estimation with the graphical lasso. Biostatistics. 2008;9(3):432–41. 18079126 10.1093/biostatistics/kxm045 PMC3019769

[26] Meinshausen N, Bühlmann P. High-dimensional graphs and variable selection with the lasso 2006.

[27] Zhao T, Liu H, Roeder K, Lafferty J, Wasserman L. The huge package for high-dimensional undirected graph estimation in r. J Mach Learn Res. 2012;13(1):1059–62. 26834510 PMC4729207

[28] Fisher TJ, Sun X. Improved stein-type shrinkage estimators for the high-dimensional multivariate normal covariance matrix. Comput Stat Data Anal. 2011;55(5):1909–18.

[29] Mar JC. The rise of the distributions: why non-normality is important for understanding the transcriptome and beyond. Biophys Rev. 2019;11(1):89–94. 30617454 10.1007/s12551-018-0494-4 PMC6381358

[30] Zwiener I, Frisch B, Binder H. Transforming RNA-Seq data to improve the performance of prognostic gene signatures. PLoS ONE. 2014;9(1):85150. 10.1371/journal.pone.0085150 PMC3885686 24416353

[31] Church BV, Williams HT, Mar JC. Investigating skewness to understand gene expression heterogeneity in large patient cohorts. BMC Bioinf. 2019;20(24):1–14. 10.1186/s12859-019-3252-0 PMC6923883 31861976

[32] Becht E, McInnes L, Healy J, Dutertre C-A, Kwok IW, Ng LG, Ginhoux F, Newell EW. Dimensionality reduction for visualizing single-cell data using UMAP. Nat Biotechnol. 2019;37(1):38–44. 10.1038/nbt.4314 30531897

[33] Liu H, Lafferty J, Wasserman L. The nonparanormal: semiparametric estimation of high dimensional undirected graphs. J Mach Learn Res. 2009;10(10) PMC4729207 26834510

[34] Strimmer K. A unified approach to false discovery rate estimation. BMC Bioinf. 2008;9(1):1–14. 10.1186/1471-2105-9-303 PMC2475539 18613966

[35] Bernal V, Soancatl-Aguilar V, Bulthuis J, Guryev V, Horvatovich P, Grzegorczyk M. Genenettools: tests for gaussian graphical models with shrinkage. Bioinformatics. 2022;38(22):5049–54. 36179082 10.1093/bioinformatics/btac657 PMC9665865

[36] Lun AT, McCarthy DJ, Marioni JC. A step-by-step workflow for low-level analysis of single-cell RNA-Seq data with bioconductor. F1000Research 2016;5. 10.12688/f1000research.9501.1 PMC5112579 27909575

[37] Chicco D, Jurman G. The advantages of the Matthews correlation coefficient (mcc) over f1 score and accuracy in binary classification evaluation. BMC Genomics. 2020;21(1):1–13. 10.1186/s12864-019-6413-7 PMC6941312 31898477

[38] Kerrien S, Aranda B, Breuza L, Bridge A, Broackes-Carter F, Chen C, Duesbury M, Dumousseau M, Feuermann M, Hinz U, et al. The intact molecular interaction database in 2012. Nucleic Acids Res. 2012;40(D1):841–6. 10.1093/nar/gkr1088 PMC3245075 22121220

[39] Szklarczyk D, Gable AL, Nastou KC, Lyon D, Kirsch R, Pyysalo S, Doncheva NT, Legeay M, Fang T, Bork P, et al. The string database in 2021: customizable protein-protein networks, and functional characterization of user-uploaded gene/measurement sets. Nucleic Acids Res. 2021;49(D1):605–12. 10.1093/nar/gkaa1074 PMC7779004 33237311

[40] Oughtred R, Rust J, Chang C, Breitkreutz B-J, Stark C, Willems A, Boucher L, Leung G, Kolas N, Zhang F, et al. The biogrid database: a comprehensive biomedical resource of curated protein, genetic, and chemical interactions. Protein Sci. 2021;30(1):187–200. 33070389 10.1002/pro.3978 PMC7737760

[41] Lock A, Rutherford K, Harris MA, Hayles J, Oliver SG, Bähler J, Wood V. Pombase 2018: user-driven reimplementation of the fission yeast database provides rapid and intuitive access to diverse, interconnected information. Nucleic Acids Res. 2019;47(D1):821–7. 10.1093/nar/gky961 PMC6324063 30321395

[42] Teixeira MC, Monteiro PT, Palma M, Costa C, Godinho CP, Pais P, Cavalheiro M, Antunes M, Lemos A, Pedreira T, et al. Yeastract: an upgraded database for the analysis of transcription regulatory networks in saccharomyces cerevisiae. Nucleic Acids Res. 2018;46(D1):348–53. 10.1093/nar/gkx842 PMC5753369 29036684

[43] Liska O, Bohár B, Hidas A, Korcsmáros T, Papp B, Fazekas D, Ari E. Tflink: an integrated gateway to access transcription factor-target gene interactions for multiple species. Database. 2022;2022:083. 10.1093/database/baac083 PMC9480832 36124642

[44] Tian J, Wang J, Roeder K. Esco: single cell expression simulation incorporating gene co-expression. Bioinformatics. 2021;37(16):2374–81. 33624750 10.1093/bioinformatics/btab116 PMC8388018

[45] Luecken MD, Theis FJ. Current best practices in single-cell RNA-Seq analysis: a tutorial. Mol Syst Biol. 2019;15(6):8746. 10.15252/msb.20188746 PMC6582955 31217225

[46] Stegle O, Teichmann SA, Marioni JC. Computational and analytical challenges in single-cell transcriptomics. Nat Rev Genet. 2015;16(3):133–45. 25628217 10.1038/nrg3833

[47] Zhang MJ, Ntranos V, Tse D. Determining sequencing depth in a single-cell RNA-Seq experiment. Nat Commun. 2020;11(1):774. 32034137 10.1038/s41467-020-14482-y PMC7005864

[48] Salehi H, Gorodetsky A, Solhmirzaei R, Jiao P. High-dimensional data analytics in civil engineering: a review on matrix and tensor decomposition. Eng Appl Artif Intell. 2023;125: 106659.

[49] Li WV, Li Y. sclink: inferring sparse gene co-expression networks from single-cell expression data. Genomics Proteomics Bioinf. 2021;19(3):475–92. 10.1016/j.gpb.2020.11.006 PMC8896229 34252628

[50] Saint M, Bertaux F, Tang W, Sun X-M, Game L, Köferle A, Bähler J, Shahrezaei V, Marguerat S. Single-cell imaging and RNA sequencing reveal patterns of gene expression heterogeneity during fission yeast growth and adaptation. Nat Microbiol. 2019;4(3):480–91. 30718845 10.1038/s41564-018-0330-4

[51] Nadal-Ribelles M, Islam S, Wei W, Latorre P, Nguyen M, Nadal E, Posas F, Steinmetz LM. Sensitive high-throughput single-cell rna-seq reveals within-clonal transcript correlations in yeast populations. Nat Microbiol. 2019;4(4):683–92. 30718850 10.1038/s41564-018-0346-9 PMC6433287

[52] Poran A, Nötzel C, Aly O, Mencia-Trinchant N, Harris CT, Guzman ML, Hassane DC, Elemento O, Kafsack BF. Single-cell RNA sequencing reveals a signature of sexual commitment in malaria parasites. Nature. 2017;551(7678):95–9. 29094698 10.1038/nature24280 PMC6055935

[53] Zeisel A, Hochgerner H, Lönnerberg P, Johnsson A, Memic F, Van Der Zwan J, Häring M, Braun E, Borm LE, La Manno G, et al. Molecular architecture of the mouse nervous system. Cell. 2018;174(4):999–1014. 30096314 10.1016/j.cell.2018.06.021 PMC6086934

[54] Hou J, Ye X, Feng W, Zhang Q, Han Y, Liu Y, Li Y, Wei Y. Distance correlation application to gene co-expression network analysis. BMC Bioinf. 2022;23(1):1–24. 10.1186/s12859-022-04609-x PMC8862277 35193539

[55] Hmisc FHJ. Harrell Miscellaneous. R package version 5.1-2, https://hbiostat.org/R/Hmisc/

[56] Marques S, Zeisel A, Codeluppi S, Van Bruggen D, Mendanha Falcão A, Xiao L, Li H, Häring M, Hochgerner H, Romanov RA, et al. Oligodendrocyte heterogeneity in the mouse juvenile and adult central nervous system. Science. 2016;352(6291):1326–9. 27284195 10.1126/science.aaf6463 PMC5221728

[57] Wang X, Choi D, Roeder K. Constructing local cell-specific networks from single-cell data. Proc Natl Acad Sci. 2021;118(51):2113178118. 10.1073/pnas.2113178118 PMC8713783 34903665

