# Supplementary material for: Shrinkage estimation of gene interaction networks in single-cell RNA sequencing data
Source: BMC Bioinformatics. 2024 Oct 26;25:339. doi: 10.1186/s12859-024-05946-9 (PMC11515282; doi:10.1186/s12859-024-05946-9)
Supplement: Supplementary file 1 — Supplementary material 1 [file 12859_2024_5946_MOESM1_ESM.pdf]

Supplementary material

Shrinkage estimation of gene interaction networks in single-cell RNA sequencing data

Duong H.T. Vo, Thomas Thorne

1 Covariance matrix shrinkage workflows

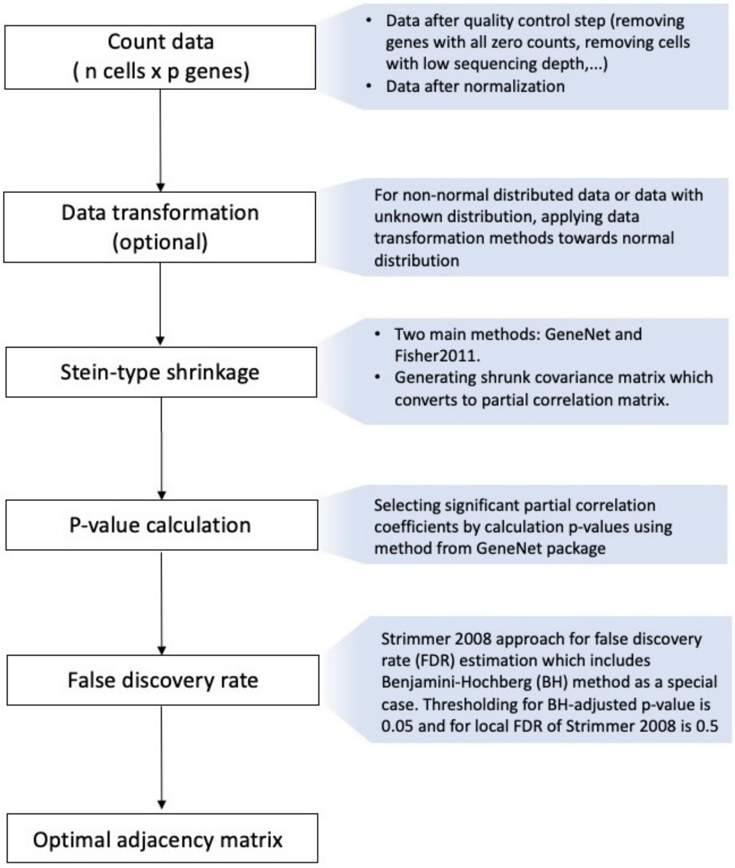

Figure S1: Covariance matrix shrinkage workflows of Stein-type shrinkage. Inverse covariance matrix shrinkage estimation starts with processing count data with data transformation. In Stein-type shrinkage workflow, GeneNet and Fisher2011 are implemented and partial correlation matrix is estimated from shrunk covariance matrix. Significant coefficients in partial correlation matrix are selected based on adjusted p-values and considered as edges in final adjacency matrix.

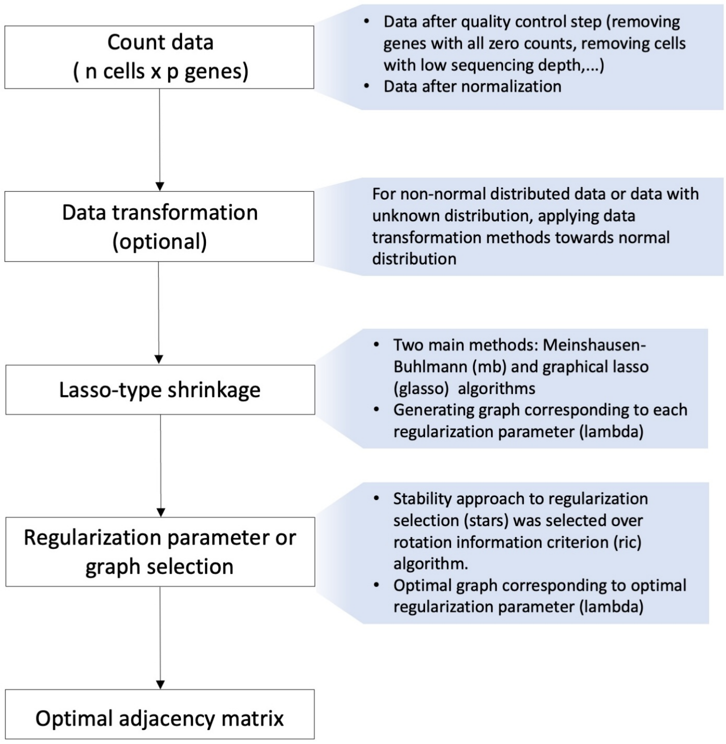

Figure S2: Covariance matrix shrinkage workflows of Lasso-type methods. Lasso-type shrinkage based on L1 regularization consists of Meinshausen-Buhlmann (mb) and graphical lasso (glasso) algorithms which generates different graphs corresponding to different regularization parameters. Optimal graph is selected by STARS algorithm [1]

2 Graph selection algorithms in Lasso-type shrinkage

In the Lasso-type shrinkage workflow, estimated adjacency matrices corresponding to each regularization parameter are produced after the shrinkage step. In terms of graph selection approaches in Lasso-type shrinkage frameworks, the STARS algorithm improves performance over the RIC algorithm when analysing in normally distributed and single-cell RNA sequencing (scRNAseq) data with mb or glasso shrinkage algorithms and no data transformation in simulation (Supplementary Figure S1 & S2). Hence, the STARS algorithm is chosen for optimal graph selection step in Lasso-type shrinkage framework[1].

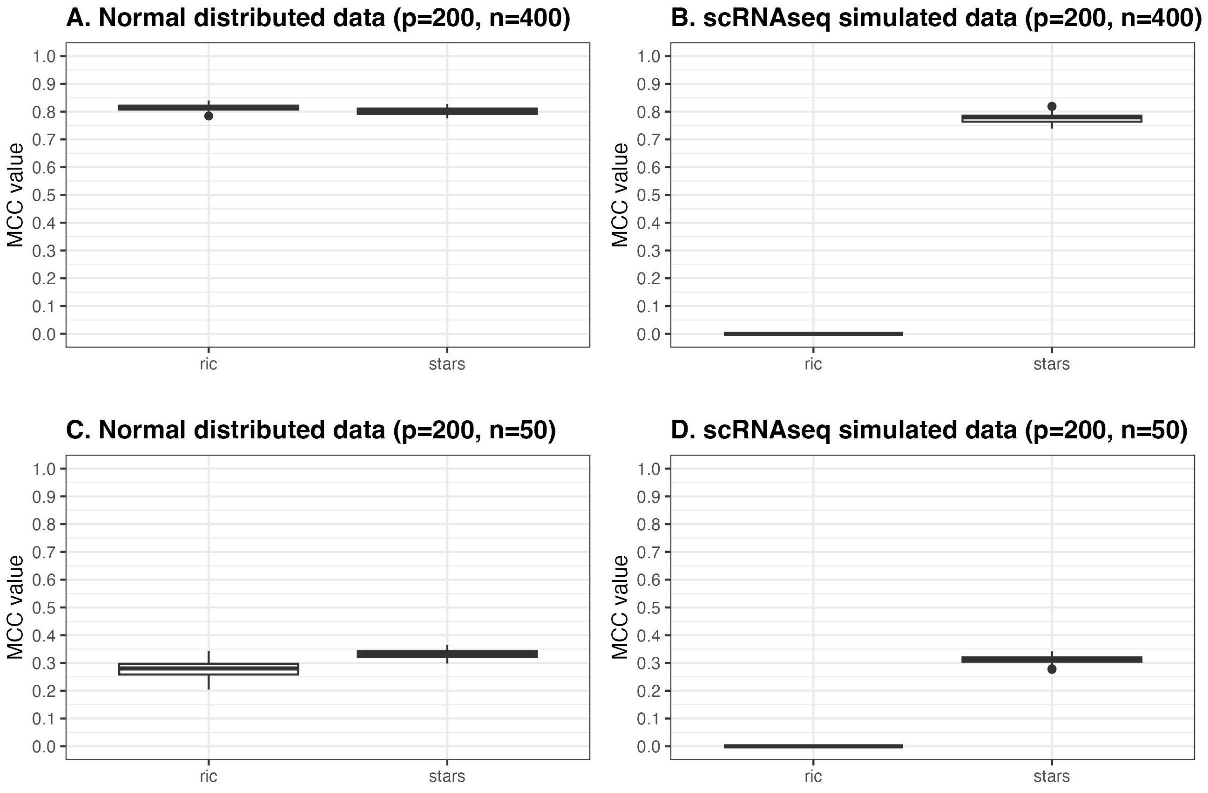

Figure S3: Comparison analysis between ric and stars algorithms in graph selection of mb shrinkage algorithm.

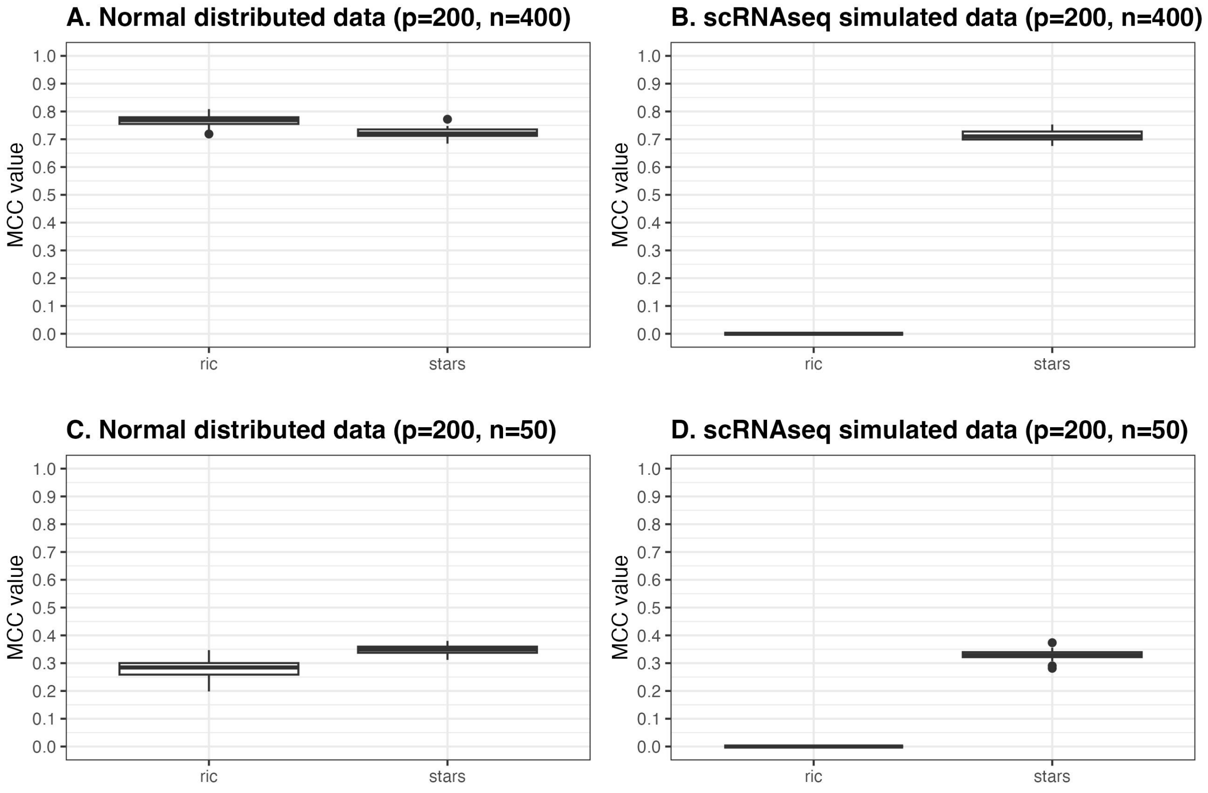

Figure S4: Comparison analysis between ric and stars algorithm in graph selection of glasso shrinkage algorithm.

3 Simulation of scRNAseq data

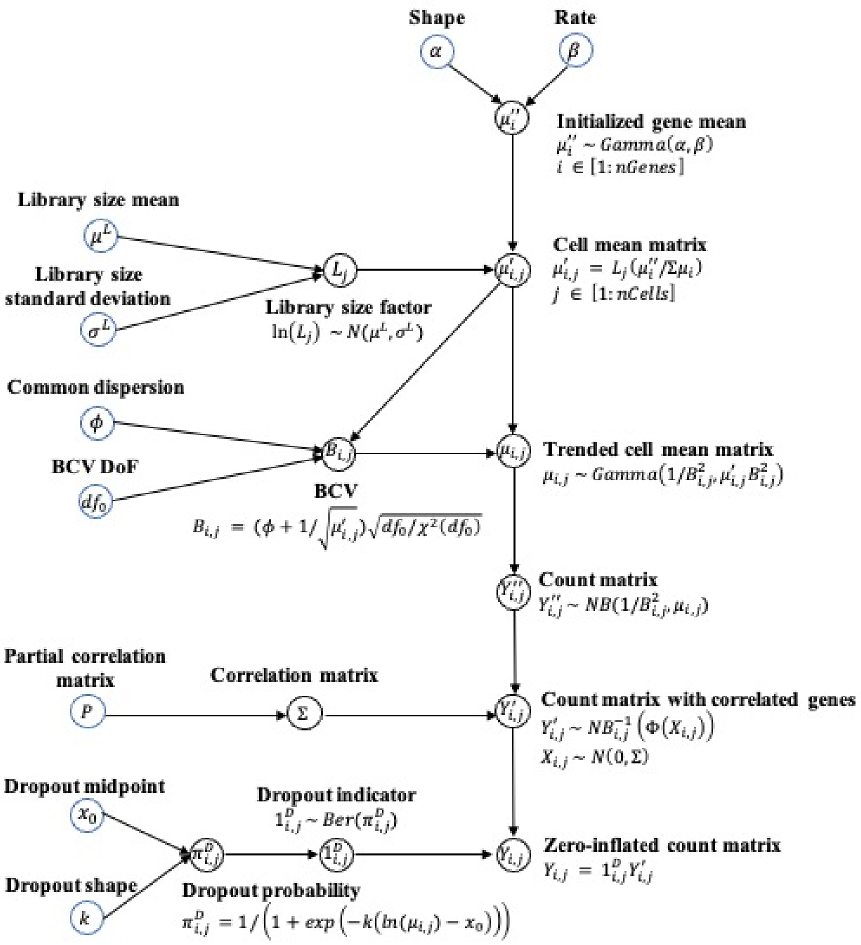

Figure S5: Simulation model. Blue circles represent input parameters whilst black circles indicate calculating or simulating steps. Simulation model is based on models from splatter and ESCO R packages [2, 3]. Gene mean is simulated from Gamma distribution. Means of gene expression are converted into matrix in which library size and Biological Coefficient of Variation (BCV) are used to adjust the expression in each cell. Final mean matrix is used to simulate count matrix from negative binomial distribution. Input partial correlation matrix is converted to correlation. Next, co-expression gene network is embedded in count matrix by using copula and dropout event is introduced through logistic expression depending on gene mean.

4 Data transformation

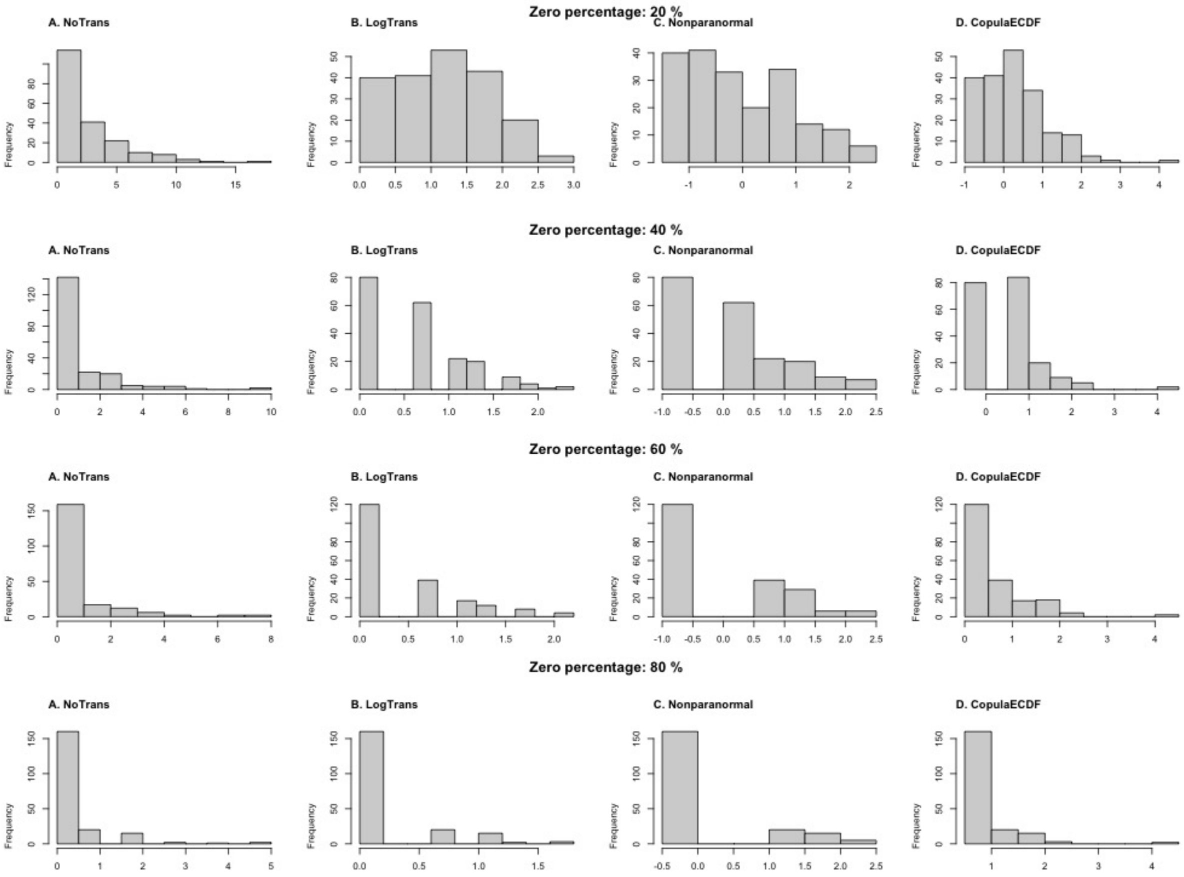

Figure S6: Histograms of 3 data transformation approaches on genes of simulated scRNAseq counts with different zero count percentage.

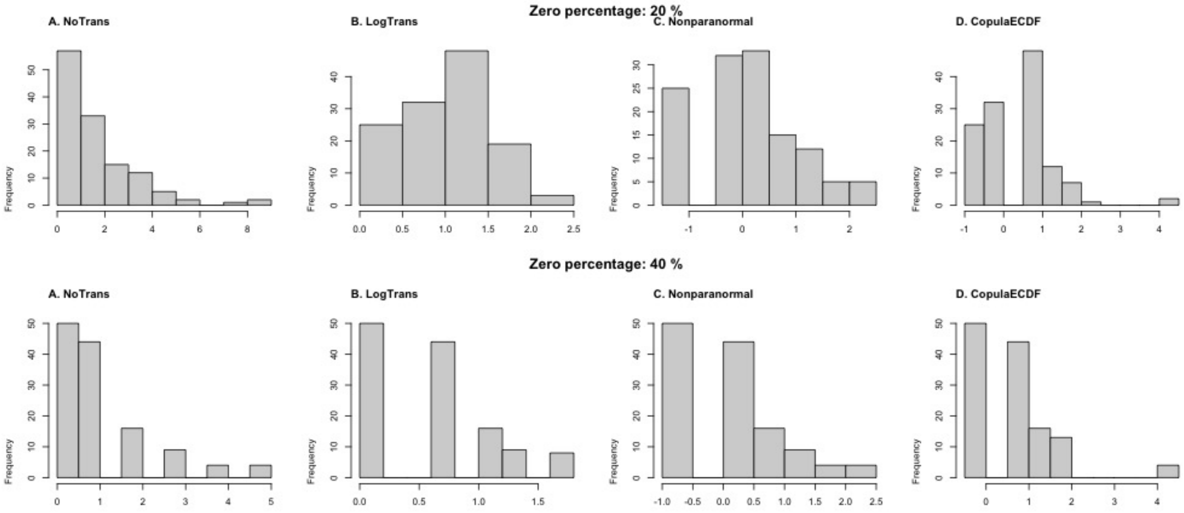

Figure S7: Histograms of 3 data transformation approaches on genes of experimental *S.cerevisiae* scRNAseq data with different zero count percentage [4].

5 Performance of Stein-type shrinkage when number of features is 2000

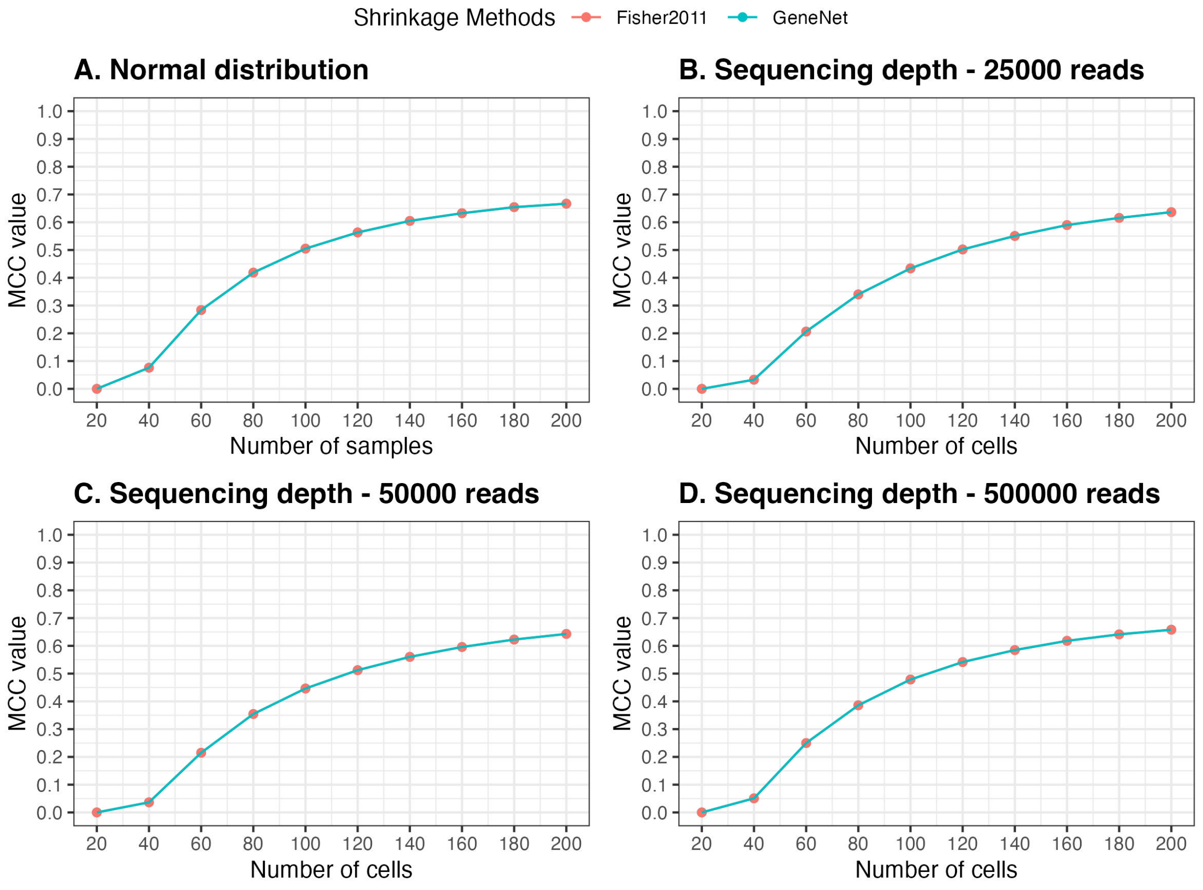

Figure S8: Performance of Stein-type shrinkage workflow when p = 2000, n = 20 to 200 and data is simulated from normal distribution (A) or scRNAseq model (B, C, D).

6 Zero proportion in experimental scRNAseq data

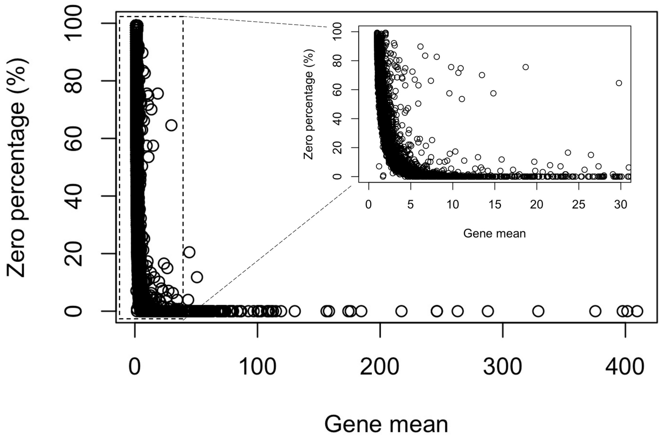

Figure S9: Zero proportion in *S.cerevisiae* scRNAseq data. Accession number: GSE122392 [4].

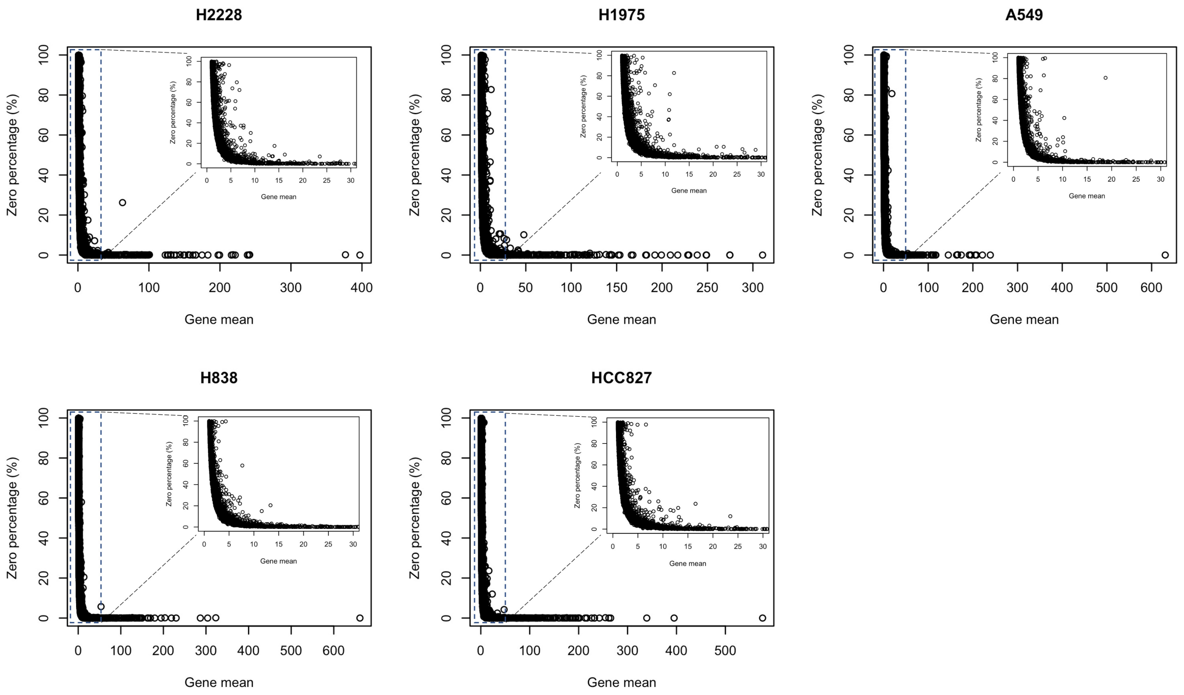

Figure S10: Zero proportion in benchmark datasets (10x Genomics protocol). Accession number: GSE118767 [5].

7 Performance of zero-inflated modelling in simulated scRNAseq data

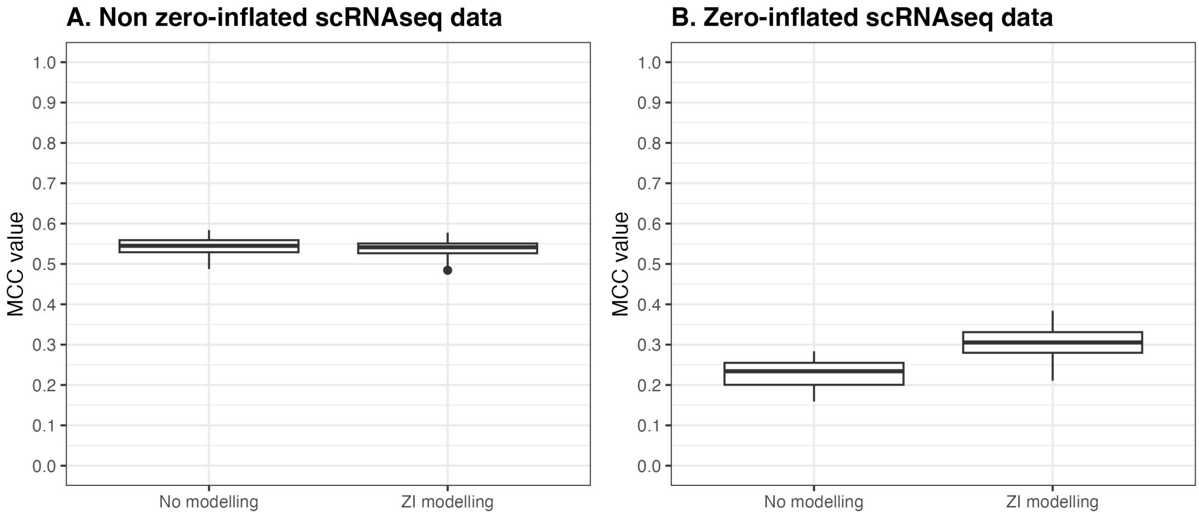

Figure S11: Performance with and without zero-inflated modelling in Stein-type shrinkage workflow in non-zero inflated (A) and zero-inflated (B) simulated scRNAseq data. (Fisher2011, p=200, n=100)

8 Performance of different gene network inference methods in 200 highly variable genes of *Saccharomyces cerevisiae* scRNAseq data

In *S.cerevisiae* scRNAseq data analysis, 200 highly variable genes are shared in all databases. Top 200 highly variable genes (HVGs) are chosen by using modelGeneCV2 and getTopHVGs functions in scran R package [4, 6]. Positive predictive rate is shown in Table **S1**. Zero-inflated GeneNet shrinkage estimates less edges, however, around 30% of estimated edges are reported in interaction databases compared to GeneNet, scLink and Pearson correlation methods (PPV $\simeq$ 12%).

Table S1: Positive predictive rates of *S.cerevisiae* scRNAseq data analysis.

Methods	Positive predictive rates
ZIGeneNet	$32/108 \simeq 29.63\%$
GeneNet	$10/78 \simeq 12.82\%$
scLink	$46/355 \simeq 12.96\%$
Pearson	$1242/10572 \simeq 11.75\%$

9 Performance of Pearson correlation method in experimental scRNAseq data analysis with thresholds

Pearson correlation matrix is calculated with a threshold value on the correlation to build the final adjacency matrix of 0.5. Results of the experiments are shown in table **S2**.

Table S2: Positive predictive rates of Pearson correlation method with thresholds in scRNAseq data analysis. Order of the analysis is the same as table 3. Threshold value is 0.5.

Data	Positive predictive rates
[7]	$40/787 \simeq 5.1\%$
[4]	$30137/283894 \simeq 10.62\%$
[8]	$14/41 \simeq 34.15\%$
[9]	$8/498 \simeq 1.61\%$

References

[1] Han Liu, Kathryn Roeder, and Larry Wasserman. "Stability approach to regularization selection (stars) for high dimensional graphical models". In: *Advances in neural information processing systems* 23 (2010).

[2] Jinjin Tian, Jiebiao Wang, and Kathryn Roeder. "ESCO: single cell expression simulation incorporating gene co-expression". In: *Bioinformatics* 37.16 (2021), pp. 2374–2381.

[3] Luke Zappia, Belinda Phipson, and Alicia Oshlack. "Splatter: simulation of single-cell RNA sequencing data". In: *Genome biology* 18.1 (2017), p. 174.

[4] Mariona Nadal-Ribelles et al. "Sensitive high-throughput single-cell RNA-seq reveals within-clonal transcript correlations in yeast populations". In: *Nature Microbiology* 4.4 (2019), pp. 683–692.

[5] Luyi Tian et al. "Benchmarking single cell RNA-sequencing analysis pipelines using mixture control experiments". In: *Nature methods* 16.6 (2019), pp. 479–487.

[6] Aaron TL Lun, Davis J McCarthy, and John C Marioni. "A step-by-step workflow for low-level analysis of single-cell RNA-seq data with Bioconductor". In: *F1000Research* 5 (2016).

[7] Malika Saint et al. "Single-cell imaging and RNA sequencing reveal patterns of gene expression heterogeneity during fission yeast growth and adaptation". In: *Nature microbiology* 4.3 (2019), pp. 480–491.

[8] Asaf Poran et al. "Single-cell RNA sequencing reveals a signature of sexual commitment in malaria parasites". In: *Nature* 551.7678 (2017), pp. 95–99.

[9] Amit Zeisel et al. "Molecular architecture of the mouse nervous system". In: *Cell* 174.4 (2018), pp. 999–1014.
